# Preconditioning of bone marrow mesenchymal stem cells with sodium hydrosulfide enhances their therapeutic potential in type II collagen–induced arthritis rat model

**DOI:** 10.1007/s00210-025-04222-8

**Published:** 2025-05-14

**Authors:** Sara M. El-Sayed, Hanaa H. Ahmed, Hadeer A. Aglan, Mohamed M. Naguib, Mohamed R. Mohamed

**Affiliations:** 1 Biochemistry Department, Faculty of Science, Ain Shams University, P.O. 11566, Cairo, Egypt; 2 Hormones Department, Medical Research and Clinical Studies Institute, National Research Centre, Giza, Egypt; 3 Stem Cells Lab, Center of Excellence for Advanced Sciences, National Research Centre, Giza, Egypt

**Keywords:** Rheumatoid arthritis, BM-MSCs, NaHS, Inflammatory mediators, Rats

## Abstract

This study was conducted to evaluate the impact of sodium hydrogen sulfide (NaHS) on the therapeutic efficacy of bone marrow mesenchymal stem cells (BM-MSCs) in the treatment of collagen-induced arthritis (CIA) rats. MSCs were isolated and cultured from rat bone marrow, and their characteristics were determined. The CIA model was induced in rats by intradermal injections of type II collagen on days 0 and 21. A variety of treatments were administered, including naproxen, BM-MSCs, BM-MSC-conditioned media, NaHS, BM-MSCs preconditioned with NaHS, and BM-MSCs preconditioned with NaHS-conditioned media. The infused BM-MSCs homed to the bone trabeculae and cartilage of the knee joint, leading to significant improvements in gait scores and a reduction in paw withdrawal frequency (PWF). Treatment with BM-MSCs and NaHS also significantly suppressed serum levels of CRP, RF, and 14-3-3η, while downregulating TNF-α gene expression and MMP-1 protein levels in the synovial membrane. Histopathological analysis confirmed these biochemical and molecular genetic findings. Notably, CIA rats treated with BM-MSCs preconditioned with NaHS showed the most significant improvements, with outcomes closely resembling those of healthy controls. This study concludes that NaHS enhances the therapeutic efficacy of BM-MSC therapy for rheumatoid arthritis (RA) by augmenting their anti-inflammatory, immunomodulatory, and regenerative properties.

## Introduction

Rheumatoid arthritis (RA) is a chronic, systemic autoimmune disorder that primarily affects the joints and their surrounding membranes, particularly in the hands and feet. It is characterized by the infiltration of immune cells, synovial hyperplasia, pannus formation, and subsequent destruction of bone and cartilage. If left untreated, RA can lead to necrosis, granulation tissue adhesion, and fibrous tissue formation on the articular cartilage surface (Makkar et al. 2023). Extensive research for the past three decades has investigated the global prevalence and incidence of (RA), demonstrating that the disease affects individuals worldwide, irrespective of race, sex, ethnicity, nationality, or age. However, its incidence and prevalence rates vary based on population characteristics and have fluctuated over time (Safiri et al. 2019). RA is more prevalent in northern regions than in southern regions and is more common in urban areas than in rural areas (Littlejohn and Monrad 2018; Smolen et al. 2016). A recent study conducted by the Egyptian College of Rheumatology found that the average age of onset for RA in Egyptian patients is 38.4 ± 11.6 years, which is significantly younger than that observed in non-Egyptian populations. The earliest onset was recorded in upper Egypt. Additionally, approximately 2% of patients experience juvenile-onset RA, with an average disease duration of 6.4 ± 6 years. Egyptian RA patients also exhibit greater functional disability and more active disease progression at younger ages compared to their non-Egyptian counterparts (Gheita et al. 2023).

Clinically, the symptoms of RA differ significantly between the early-stages and advanced inadequately treated later stages. In the early stages, patients often present with generalized symptoms, including joint swelling and tenderness, flu-like sensations, fatigue, and morning stiffness. These symptoms are typically associated with elevated C-reactive protein (CRP) levels and an increased erythrocyte sedimentation rate (ESR) (Brzustewicz et al. 2017). In contrast, untreated or poorly managed RA leads to severe systemic complications, including lung nodules, interstitial lung disease, pleural effusions, lymphomas, vasculitis affecting small or medium-sized arteries, atherosclerosis, hematologic abnormalities, keratoconjunctivitis, and lymphomas. Additionally, progressive joint damage results in malalignment, reduced range of motion, cartilage destruction, bone erosion, and rheumatic nodules. These complications, driven by chronic systemic inflammation, significantly contribute to increased mortality rates in RA patients (Aletaha and Smolen 2018; Littlejohn and Monrad 2018).

Current therapeutic strategies for RA mainly focus on delaying disease progression by controlling inflammation (Firestein and McInnes 2017). Methotrexate (MTX) remains the first-line treatment; however, approximately 30–50% of patients develop resistance to MTX over time (Escal et al. 2025). Advances in RA research have led to the development of biologic agents, including tumor necrosis factor-α (TNF-α) inhibitors and interleukin-6 (IL-6) receptor antagonists, which have shown efficacy in disease management (Gao et al. 2024). Furthermore, Janus kinase (JAK) inhibitors, such as upadacitinib (Rinvoq), baricitinib (Olumiant), and tofacitinib (Xeljanz), have been introduced as targeted synthetic disease-modifying antirheumatic drugs (DMARDs) offering new options for patients unresponsive to traditional therapies (Angelini et al. 2020). Despite these advancements, a significant proportion of patients with moderate-to-severe RA exhibit inadequate responses to current treatments, highlighting the urgent need for novel therapeutic approaches that offer enhanced efficacy and fewer adverse effects.

Bone marrow mesenchymal stem cells (BM-MSCs) are stromal cells within the bone marrow that provide mechanical support for hematopoietic stem cells (HSCs) and also release various growth factors, such as leukemia inhibitory factor (LIF), interleukin-6 (IL-6), IL-11, macrophage-colony stimulating factor (M-CSF), and stem cell factor (SCF), to support hematopoiesis (Ohishi and Schipani 2010). Previous studies have shown that BM-MSCs also play a role in regulating immune responses, including suppressing T lymphocyte proliferation and inhibiting the production of inflammatory cytokines (Liu et al. 2015; Sánchez-Berná et al. 2015). These abilities make BM-MSCs a potential treatment for various autoimmune diseases, such as RA (Qiao and Ma 2009), multiple sclerosis (Yamout et al. 2010), systemic lupus erythematosus (Vagnani et al. 2015), and Crohn’s disease (Dave et al. 2016). Numerous studies have demonstrated the therapeutic potential of BM-MSC transplantation for treating RA (Sarsenova et al. 2021b, a; Li et al. 2024). To enhance the therapeutic effectiveness of BM-MSCs in RA, researchers have explored various strategies, including modifying the injection site and combining BM-MSCs with other therapeutic agents. However, there is limited research on using genetic engineering methods to enhance the success of BM-MSCs in treating RA (Tian et al. 2019).

Hydrogen sulfide (H_2_S) has been emerged as a novel gasotransmitter signaling molecule within the central nervous system (CNS), playing a crucial role in regulating ion channels, neurotransmitter functions, and other intracellular signaling molecules including tyrosine kinases (Wang 2012). Growing evidence suggests that exogenous H_2_S acts as a powerful neuroprotective agent through its anti-inflammatory, antioxidant, and anti-apoptotic properties (Feng et al. 2014; Hu et al. 2007; Kimura 2013). Additionally, H_2_S has been shown to enhance the proliferation and neuronal differentiation of neural stem cells, as well as protect against hypoxia-induced inhibition of hippocampal neurogenesis (Liu et al. 2014). While there is limited information regarding the anti-apoptotic impact of endogenous H_2_S on BM-MSCs, the potential therapeutic value of H_2_S for BM-MSC transplantation is gaining increasing recognition (Guo et al. 2015).

The rationale of this study was to evaluate the effectiveness of NaHS, a hydrogen sulfide (H_2_S) donor, in enhancing BM-MSC therapy for rheumatoid arthritis in an experimental rat model. To achieve this goal, relevant biochemical markers were assessed using ELISA, real-time qPCR, and Western blot techniques.

## Materials and methods

### In vitro protocol

#### Isolation, propagation, and characterization of BM-MSCs

Bone marrow cells were isolated from 6-week-old male albino *Wistar* rats by flushing the femoral and tibial medullary cavities with Dulbecco’s modified Eagle’s medium (DMEM)-high glucose (Biowest, France), supplemented with 30% fetal bovine serum (FBS Biowest). Mononuclear cells were purified using density gradient centrifugation at 400 × g for 30 min. After three washes with phosphate-buffered saline (PBS, Biowest), the purified cells were cultured into 25-cm^2^ cell culture flasks containing complete culture medium (DMEM-high glucose with 30% FBS and 1% penicillin/streptomycin, Biowest) and maintained at 37 °C in a humidified incubator with 5% CO_2_. The culture medium was replaced with fresh medium after 48 h and non-adherent cells were discarded. The adherent cells, characterized as BM-MSCs, were cultured in complete medium upon reaching 80–90% confluence, labelled as passage zero (P0) cells. P0 cells were rinsed with PBS and collected using a 0.25% trypsin–EDTA solution (Biowest) for 5 min at 37 °C. The detached cells were centrifuged at 200 × g for 10 min, resuspended in complete culture medium, counted, and re-plated as passage one (P1) at a density of 1 × 10⁶ cells per flask. The medium was refreshed every 3 days for a 10–14-day period. The same protocol was followed for each passage, and cells were harvested and passaged by trypsinization upon reaching 80–90% confluence (Alhadlaq and Mao 2004).

To characterize the cultured BM-MSCs, the cells were observed under an inverted optical microscope (Olympus, Japan) to confirm their typical fibroblast-like spindle shape. In the third passage, the cells underwent immunophenotyping to assess the expression of different cell surface antigens. The cells were harvested via trypsinization, washed with PBS, and aliquoted at a concentration of 0.5 × 10⁶ cells/mL. They were then stained for 30 min at room temperature in the dark with monoclonal antibodies, including phycoerythrin-conjugated CD34^−^, CD44^+^, and CD73^+^ (Invitrogen, Thermo Fisher Scientific, Waltham, MA, USA). After staining, the cells were washed twice with PBS, resuspended in PBS, and analyzed using a COULTER EPICS XL flow cytometer equipped with SYSTEM II software (Beckman Coulter, Brea, CA, USA), following the manufacturer’s protocol (Dominici et al. 2006; Aglan et al. 2024a, 2024b).

#### Preconditioning of BM-MSCs with NaHS

The third passage of BM-MSCs was co-cultured with 200 μmol/L NaHS (Sigma-Aldrich, St. Louis, MO, USA) for 30 min, as previously described by Xie et al. (2012), before being transplanted into CIA rats.

#### Generation of MSCs conditioned medium

Third-passage BM-MSCs and BM-MSCs preconditioned with NaHS at 80–90% confluence were washed twice with PBS and cultured in FBS-free DMEM for 24 h. The medium from an equal number of cells (3 × 10^6^ cells) in each culture was collected and centrifuged at 400 × g for 20 min to prepare the conditioned medium (Ionescu et al. 2012).

#### Labeling of BM-MSCs and BM-MSCs preconditioned with NaHS

Third-passage BM-MSCs and BM-MSCs preconditioned with NaHS were incubated for 24 h with a ferumoxide injectable solution (Feridex IV, Berlex Laboratories, Cedar Knolls, NJ, USA) and poly-L-lysine (PLL, Sigma-Aldrich) at final concentrations of 25 μg/mL and 375 ng/mL, respectively. Prior to incubation, ferumoxide was mixed with PLL in a 1:10 ratio and shaken for 30 min at room temperature. The mixture was then added to the supplemented medium, which consisted of DMEM-high glucose supplemented with FBS, 100 U/mL penicillin, and 100 μg/mL streptomycin. Prussian blue staining and eosin counterstaining were used to track the iron particles in ferumoxide-labeled BM-MSCs in knee joint sections, verifying their homing into the knee joints (Balakumaran et al. 2010; Abdel Halim et al. 2021).

### In vivo design

#### Animals and treatments

Sixty-four adult male albino Wistar rats weighing between 140 and 160 g were obtained from the Animal Care Unit at the National Research Centre in Giza, Egypt. The rats were housed in polypropylene cages in a well-ventilated room with an ambient temperature of 25 ± 1 °C and a relative humidity of 60 ± 5%. They were kept on a 12-h light/dark cycle and had ad libitum access to tap water and a standard rodent chow. The chow consisted of 10% casein, 4% salt mixture, 1% vitamin mixture, 10% corn oil, 5% cellulose, and corn starch, making up 100 g of the diet (Meladco Co., Cairo, Egypt). Prior to the start of the experiment, the rats were given a 1-week acclimatization period.

#### Ethical approval

All animal experiments were carried out in compliance with the ethical guidelines for the experimental animals and got the approval of the Medical Research Ethics Committee of the National Research Center (NRC), Giza, Egypt (Approval No. 20 062), complying with the ARRIVE guidelines and was conducted according to the eighth edition (2011) guidelines of the National Institutes of Health (NIH) for the Care and Use of Experimental Animals (NIH publications No.8023, revised 1978).

#### Creation of CIA model

The rats were injected intradermally into the base of the tail with 1.5 mg of bovine type II collagen (COL-II)-CFA emulsion. The collagen solution was prepared by dissolving type II collagen (obtained from Beijing SEMNL Biotechnology Co., Ltd, Beijing, China) in 0.1 M acetic acid and emulsified in complete Freund’s adjuvant (CFA) (containing Mycobacterium tuberculosis purchased from Sigma-Aldrich) at a ratio of 1:1 (v/v). A second booster injection of 750 μg of COL-II in incomplete Freund’s adjuvant (ICFA) purchased from Sigma-Aldrich at a ratio of 1:1 (v/v) was administered on day 21 post-immunization to enhance the immune response (Park et al. 2016).

#### Animal grouping

Rats (*n* = 64) were randomly allocated into 8 groups (8 rats/group). Group 1 served as health control group and received a single intradermal injection of 1.5 mL of acetic acid (as a first dose) and 0.75 mL 3 weeks later (as a booster dose) (control). The rats in Groups 2–8 were subjected to arthritis induction. One month following the induction of arthritis, the rats in Group 2 were left untreated and served as the diseased control (CIA untreated). Group 3 received oral administration ensures sustained systemic exposure of naproxen (10 mg/kg b.wt.) in 1 mL of saline once daily for 2 months (Comi et al. 2017) (CIA + naproxen). Group 4 received a single intravenous infusion of BM-MSCs (5 × 10^6^ cells/rat) in 0.5 mL of PBS (Haikal et al. 2019) (CIA + BM-MSCs). Group 5 received a single intravenous infusion of 0.5 mL of BM-MSCs conditioned media (CIA + BM-MSCs-CM). Group 6 received daily intraperitoneal injections of NaHS (1.4 µmol/kg) in 1 mL of saline for 2 months (Fang et al. 2009) (CIA + NaHS). Group 7 received a single intravenous infusion of BM-MSCs preconditioned with NaHS (5 × 10^6^ cells/rat) in 0.5 mL of PBS (CIA + BM-MSCs-NaHS). Group 8 received a single intravenous infusion of 0.5 ml of BM-MSCs preconditioned with NaHS-conditioned media (CIA + BM-MSCs-NaHS-CM).

## Valuation of the therapeutic efficacy of suggested treatments against CIA

### Behavioral examinations

#### Gait score

The assessment of knee joint pain involved evaluating animal behavior through a modified gait score, which was based on the rats’ walking pattern. Each rat was placed on an open bench in a quiet, dimly lit room and allowed to walk freely. The severity of walking disturbances was graded. A scale of 0–3 with 0 indicating normal behavior, 1 representing mild disability, 2 indicating difficulty in walking due to intermittent loading of the inflamed joints, and 3 suggesting severe impairment with the rat standing on only three paws, indicating total joint immobility (Ekundi-Valentim et al. 2010).

#### Pin prick test

Rats were assessed for pain behaviors using the pin prick test, serving as an indirect measure of knee monoarthritis. The rats were housed in specialized cages with metal mesh floors, allowing access to the plantar surface of the hindpaw. During the pin prick test the plantar surface of the hindpaw was pressed with the tip of a safety pin, with sufficient intensity to elicit a reflex withdrawal response in a normal animal, without penetrating the skin. Mechanical stimuli were applied ten times to the arthritic limb through the wire mesh floor. The frequency of paw withdrawal across these trials was expressed as the percent withdrawal frequency (PWF%), calculated using a specific formula: PWF% = (number of paw withdrawals/number of trials) × 100. Avoidance behaviors, including lifting, shaking, licking the paw, or running away were considered positive indicators of pain sensitivity (Park et al. 2006).

After completing the experiment, the rats were sacrificed by cervical dislocation under full anesthesia by intraperitoneal injection of ketamine (90 mg/kg) and xylazine (5 mg/kg) (Othman et al. 2021). Blood samples were drawn from the tail vein, and serum was separated by centrifugation at 2000 × g for 10 min in a refrigerated centrifuge and then stored at − 20 °C for future analysis. Both knee joints were excised; the right knee was snap-frozen in liquid nitrogen and stored at − 80 °C for subsequent analysis of gene and protein expression, while the left knee was fixed in 10% neutral-buffered formalin solution for 24 h for histological investigation.

### Biochemical assay

#### Serological evaluation for CRP, RF, and 14-3-3 η by ELISA

The levels of serum C-reactive protein (CRP) were determined using a commercially available “Sandwich” enzyme-linked immunosorbent assay (ELISA) kit from CLOUD-CLONE CORP in Katy, TX, USA, following the instructions provided by manufacturer. Similarly, the levels of serum rheumatoid factor (RF) were measured using a “Sandwich” ELISA kit from CUSABIO in Houston, TX, USA, according to the specification provided by the manufacturer. Additionally, the levels of serum 14-3-3 η protein were assessed using a “competitive” ELISA kit from MyBioSource in San Diego, CA, USA, following the guidance provided by manufacturer.

#### RT-qPCR analysis for TNF-α in synovial membrane

Tumor necrosis factor alpha (TNF-α) gene expression levels were assessed in the synovial membrane of knee tissues using real-time quantitative polymerase chain reaction **(**RT-qPCR). Total RNA from the synovial membrane was purified using RNeasy purification reagent (Qiagen, Valencia, CA, USA) following the manufacturer’s instructions. RNA integrity was detected by NanoDrop 2000 (Thermo Fisher Scientific, Rockford, IL, USA) with a ratio of 260/280 nm. Complementary DNA (cDNA) was synthesized by a high-capacity cDNA synthesis kit (Thermo Fisher Scientific, Waltham, MA, USA) following the manufacturer’s manual. Quantitative measurement of TNF-α gene expression levels were done by the PowerUp^TM^ SYBR Green PCR kit (Thermo Fisher Scientific) according to the manufacturer’s instructions, on a QuantStudio™ 3 Real-Time PCR System (Thermo Fisher Scientific). The reaction mixture (25 μL) consisted of 12.5 μL of QuantiNova SYBR Green PCR kit, 0.75 μL each of forward and reverse primers for the TNF-α gene (Eurofins, Anzinger Str, Ebersberg, Germany), 100 ng of cDNA template, and RNase-free water. Relative gene expression was determined using the comparative Ct method (2^−ΔΔCt^) (Livak and Schmittgen 2001), and β-actin was used as the endogenous control.

PCR cycling conditions were as follows: initial denaturation at 94 °C for 15 min, denaturation at 94 °C for 15 s for 40 cycles, annealing at 55 °C and extension at 72 °C for 30 s. The primer sequences for the genes are detailed in Table 1.

**Table 1:** Sequences of forward and reverse primers of β-actin and TNF-α genes for q-PCR

Gene	Primer sequence (5′ → 3′)
β-actin	Forward: CCCATCTATGAGGGTTACGC Reverse: TTTAATGTCACGCACGATTTC
TNF-α	Forward: AACTCCCAGAAAAGCAAGCA Reverse: CGAGCAGGAATGAGAAGAGG

### Western blot assay for MMP-1 protein in synovial membrane

#### Sample preparation and protein extraction

Synovial membrane tissues from the knee were collected to measure the amount of matrix metalloproteinase 1 (MMP-1) protein levels by Western blot technique. Briefly, ice-cold radioimmunoprecipitation assay (RIPA) lysis buffer was used to extract proteins from the tissues. The protein concentration was determined using the Bradford protein assay kit ((SK3041, Bio Basic Inc., Markham, Ontario, L3R 8 T4, Canada), following the manufacturer’s guidelines. Each sample, containing 20 μg of total protein was mixed with an equal volume of 2 × Laemmli buffer. Samples were boiled at 95 °C for 5 min to denature the proteins. The denatured protein samples were loaded onto TGX Stain-Free FastCast acrylamide gels (SDS-PAGE) from Bio-Rad Laboratories (TNC, USA), prepared according to the manufacturer’s protocol. Then, proteins were transferred from the gel to polyvinylidene fluoride (PVDF) membranes using the Bio-Rad Trans-Blot Turbo instrument. Membranes were blocked with 3% bovine serum albumin (BSA) in Tris-buffered saline with 0.1% Tween-20 (TBST) for 1 h at room temperature. Membranes were incubated with rabbit anti-rat MMP-1 antibody (1:2000) and mouse anti-rat β-actin antibody (1:2000), both from Thermo Fisher Scientific. After incubation, the membranes were washed 3–5 times with TBST. Membranes were then incubated with horseradish peroxidase–conjugated secondary antibodies rabbit anti-rat IgG (1:2000) (Thermo Fisher Scientific) for 1 h at room temperature. The blots were washed again with TBST 3–5 times.

The visualization and analysis were performed as follows: chemiluminescent signals were detected using Clarity Western ECL substrate from Bio-Rad. Equal volumes of Clarity Western luminol/enhancer solution (solution A) and peroxidase solution (solution B) were mixed and applied to the blot. Signals were captured using a CCD camera-based imager. Band intensities were analyzed using image analysis software, normalized against β-actin, on the Chemi Doc MP imager.

#### Histological procedure

After being fixed in 10% neutral buffered formalin for 24 h, rat knee joints were decalcified using formic acid. The specimens were then washed with tap water and dehydrated using graded ethanol series (30%, 70%, and 100%). Next, the specimens were cleared with xylene and embedded in paraffin at 56 °C in a hot air oven for 24 h. The paraffin-embedded tissue blocks were then sectioned at a thickness of 4 micron using a rotary microtome (LEITZ, Marshall Scientific, Australia). The tissue sections were collected on glass slides, deparaffinized, and stained with hematoxylin and eosin (H&E) (Sigma-Aldrich). The histopathological alterations of the knee joint were visualized using a light electric microscope (Olympus BX51 microscope, Shinjuku, Tokyo, Japan) (Stevens and Bancroft 1996).

#### Statistical processing

The experimental results were expressed as arithmetic means accompanied by their standard errors (S.E). Statistical analysis of the data was performed using one-way analysis of variance (ANOVA) with SPSS 23. Post-hoc analysis was conducted using the least significant difference (LSD) method to assess the significance between groups. Statistically significant differences were considered at *P* < 0.05.

## Results

### BM-MSC morphology

**Fig. 1:**
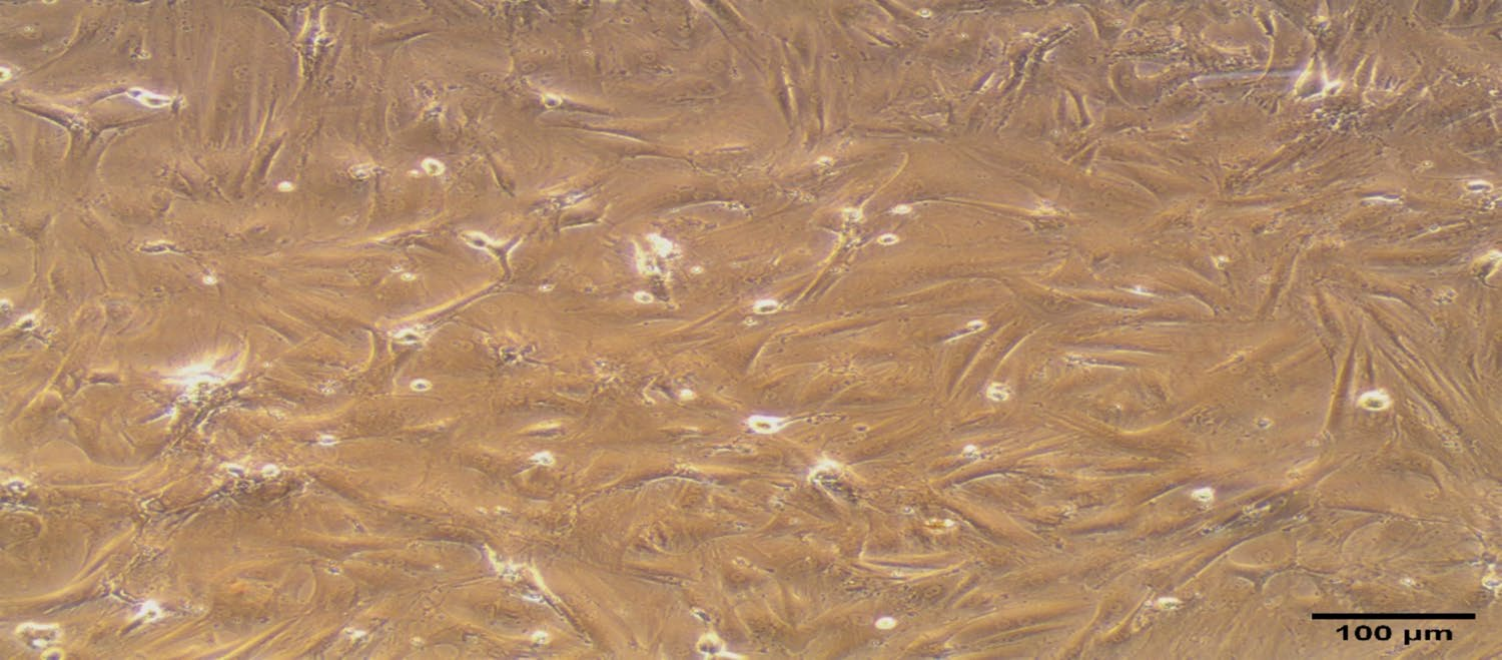
Morphological aspect of grown rat BM-MSCs at the 3^rd^ passage

The morphology of 3^rd^ passage BM-MSC population is demonstrated in the photomicrograph presented in Fig. 1. The cells have a distinct spindle-shaped appearance with a fibroblastic-like morphology.

### BM-MSC phenotyping

**Fig. 2:**
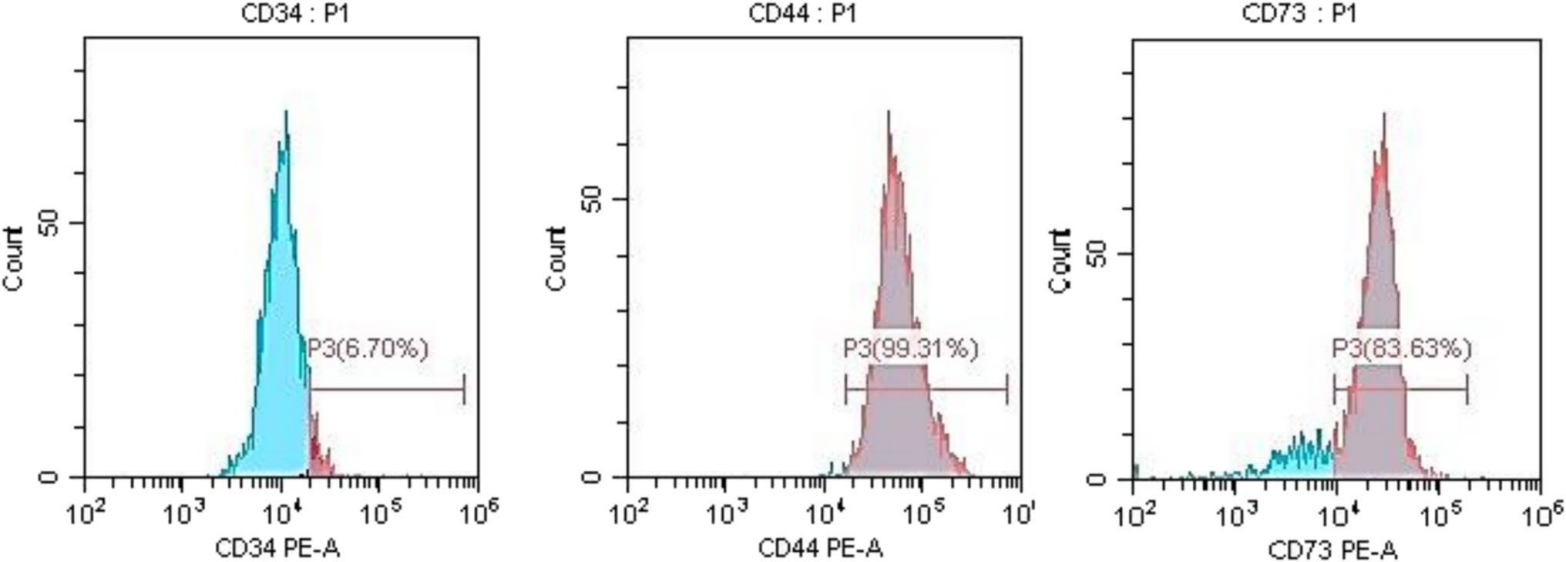
Flow cytometric analysis of MSCs surface profile of rat BM-MSCs

Flow cytometric analysis of MSC surface markers was conducted to verify that BM-MSCs retained their phenotype after culture and expansion. The results revealed that the grown BM-MSCs are positive for CD 44 (99.31%) and CD 73 (83.63%) but negative for CD 34 (6.70%) (Fig. 2).

### Homing of infused BM-MSCs and BM-MSCs preconditioned with NaHS

**Fig. 3:**
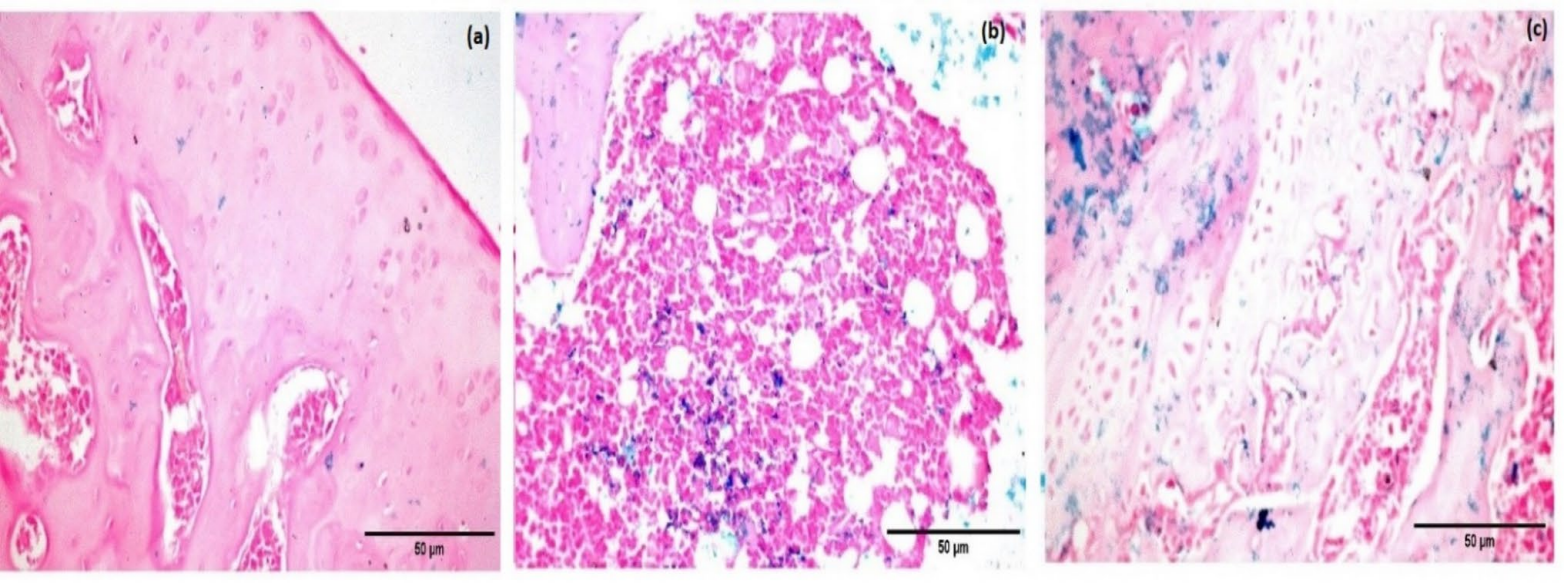
Cross section of Prussian blue stained knee joint tissues. **a** CIA group treated with unlabeled BM-MSCs, **b** CIA group treated with ferumoxides-labeled BM-MSCs, and **c** CIA group treated with ferumoxides-labeled BM-MSCs preconditioned with NaHS (scale bar 50 μm)

To ensure the homing of infused BM-MSCs and BM-MSCs preconditioned with NaHS into the knee joints of CIA model rats, the cells were labeled with ferumoxides prior to injection. The photomicrograph of the cross-sectioned rat knee joint tissue in the CIA group treated with unlabeled BM-MSCs showed a negative reaction for Prussian blue staining in the cartilaginous surface and synovial membrane (Fig. 3a). However, the photomicrographs of the cross-sectioned rat knee joint tissue in the CIA group treated with ferumoxides-labeled BM-MSCs demonstrated positive reactions for Prussian blue staining in the bone marrow and bone trabeculae (Fig. 3b). Additionally, the photomicrographs of the cross-sectioned rat knee joint tissue in the CIA group treated with or ferumoxides-labeled BM-MSCs preconditioned with NaHS demonstrated positive reactions for Prussian blue staining in the bone trabeculae and cartilage (Fig. 3c).

### Gait score and pain behavior

**Fig. 4:**
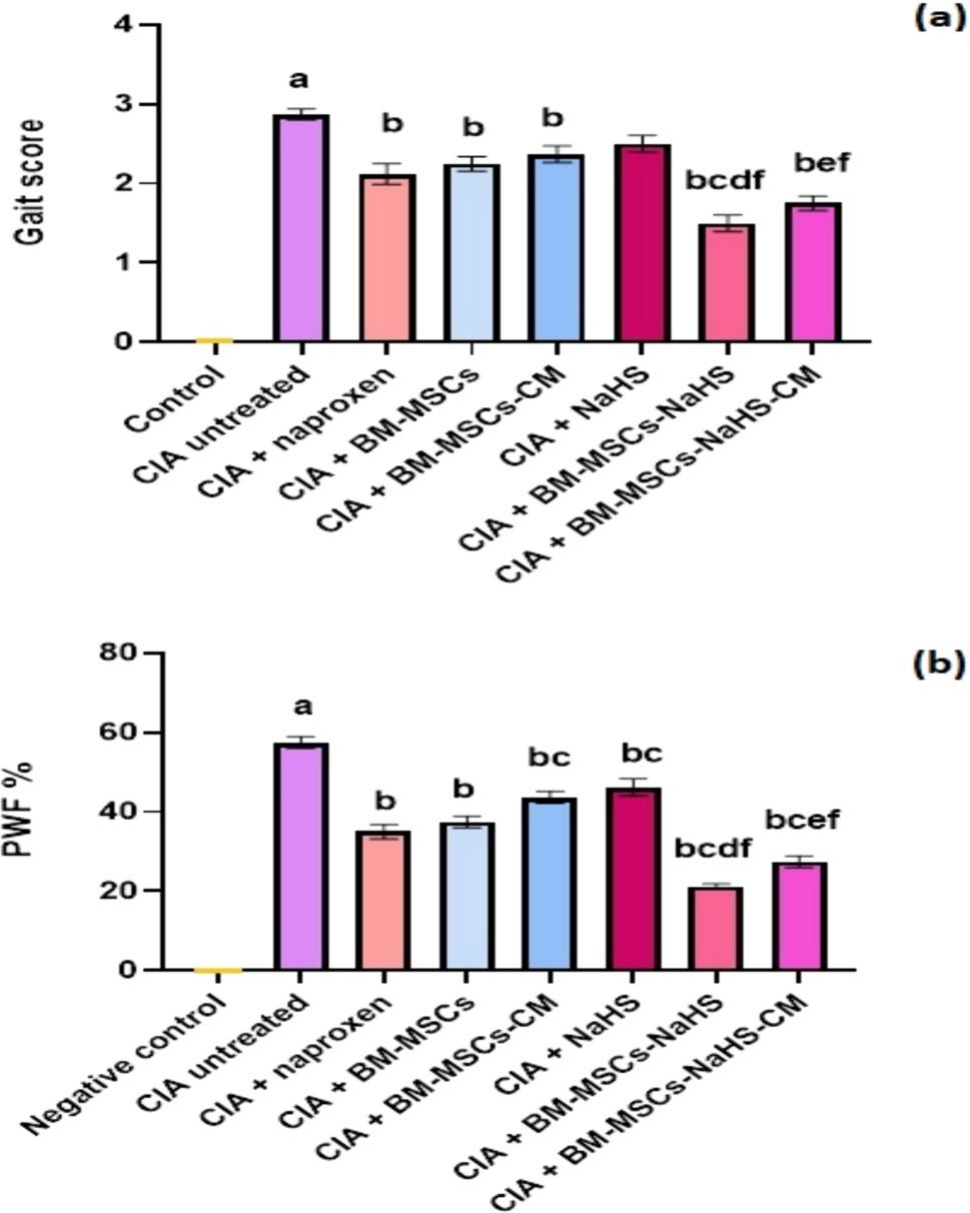
Effect of different treatments on **a** gait score and **b **paw withdrawal frequency (PWF) in CIA rats. Data are represented as means ± S.E of 8 rats/group. ^a^Significant change at *P* <0.05 in comparison with the control group. ^b^Significant change at *P* <0.05 in comparison with the untreated CIA group. ^c^Significant change at *P* <0.05 in comparison with the CIA + naproxen group. ^d^Significant change at *P* <0.05 in comparison with the CIA + BM-MSCs group. ^e^Significant change at *P* <0.05 in comparison with the CIA + BM-MSCs-CM group. ^f^Significant change at *P* < 0.05 in comparison with the CIA + NaHS group

Figure 4 illustrates the impact of various treatments on the gait score and pain behavior of CIA rats. The injection of collagen type II significantly (*P* > 0.05) impaired the normal walking pattern (as indicated by a significant elevation in gait score) and increased PWF in rats compared to the control group. However, treatment with naproxen, BM-MSCs, BM-MSCs-CM, BM-MSCs-NaHS, or BM-MSCs-NaHS-CM resulted in a significant reduction in gait score compared to untreated CIA rats. Notably, CIA rats treated with BM-MSCs-NaHS displayed a significant (*P* > 0.05) drop in gait score contrary to the CIA rats treated with naproxen, BM-MSCs, or NaHS. Similarly, treatment of CIA rats with BM-MSCs-NaHS-CM led to a significant (*P* > 0.05) decrease in gait score compared to CIA rats treated with BM-MSCs-CM or NaHS.

The treatment of CIA rats with the suggested treatments resulted in a significant (*P* > 0.05) decline in PWF compared to untreated CIA rats. Additionally, CIA rats treated with BM-MSCs-CM or NaHS displayed a significant (*P* > 0.05) elevation in PWF *versus* those treated with naproxen. In contrast, CIA rats treated with BM-MSCs-NaHS or BM-MSCs-NaHS-CM showed a significant (*P* > 0.05) depletion in PWF compared to those treated with naproxen. Interestingly, infusion of BM-MSCs-NaHS in CIA rats resulted in a significant (*P* > 0.05) drop in PWF compared to those treated with BM-MSCs or NaHS. Similarly, infusion of BM-MSCs-NaHS-CM in CIA rats caused a significant (*P* > 0.05) decline in PWF relative to those treated with BM-MSCs-CM or NaHS.

### Serological markers’ data

The data in Table 2 depicted the influence of different treatments on the serum levels of CRP, RF, and 14-3-3η in CIA rats. The successful of CIA model was achieved as indicated by the significant (*P* > 0.05) elevation of serum CRP, RF, and 14-3-3η levels in the untreated CIA rats in comparison with the controls. However, the treatment of CIA rats with the proposed treatments elicited significant (*P* > 0.05) reduction in serum CRP, RF, and 14-3-3η levels in comparison with untreated CIA rats. The group of CIA rats infused with BM-MSCs revealed significant (*P* > 0.05) elevation in serum CRP and 14-3-3η levels relative to those treated with naproxen. Likewise, in comparison with the CIA rats treated with naproxen, the groups of CIA rats treated with BM-MSCs-CM or NaHS demonstrated significant (*P* > 0.05) increase in serum CRP, RF, and 14-3-3η levels. On the opposite side, infusion of BM-MSCs-NaHS in CIA rats experienced significant (*P* > 0.05) drop in serum CRP, RF, and 14-3-3η levels compared to those treated with naproxen, BM-MSCs, or NaHS. At the same time, infusion of BM-MSCs-NaHS-CM in CIA rats produced significant (*P* > 0.05) decline in serum CRP, RF, and 14-3-3η levels *versus* those treated with BM-MSCs-CM or NaHS. Lastly, the CIA rats infused with BM-MSCs-NaHS-CM evidenced significant (*P* > 0.05) reduction in serum 14-3-3η level relative to the CIA rats treated with naproxen.

**Table 2:** Effect of different treatments on serum CRP, RF and 14-3-3η levels in CIA rats

Groups	Parameters		
	CRP (ng/mL)	RF (mIU/mL)	14-3-3 ɳ (ng/mL)
**Control**	**342.8 ± 15.3**	**43.9 ± 2.2**	**65.6 ± 1.9**
**CIA untreated**	**697.3 ± 6.9**^**a**^	**184.6 ± 3.5**^**a**^	**293.4 ± 2.5**^**a**^
**CIA + naproxen**	**444.5 ± 2.3**^**b**^	**81.4 ± 2.2**^**b**^	**135.7 ± 2.9**^**b**^
**CIA + BM-MSCs**	**495.7 ± 1.9**^**bc**^	**87.5 ± 2.7**^**b**^	**147.3 ± 1.5**^**bc**^
**CIA + BM-MSCs-CM**	**502.4 ± 2.5**^**bc**^	**89.5 ± 2.4**^**bc**^	**182.7 ± 4.0**^**bc**^
**CIA + NaHS**	**562.4 ± 7.6**^**bc**^	**92.8 ± 2.7**^**bc**^	**194.1 ± 1.6**^**bc**^
**CIA + BM-MSCs-NaHS**	**394.6 ± 4.8**^**bcdf**^	**67.9 ± 1.1**^**bcdf**^	**102.7 ± 1.3**^**bcdf**^
**CIA + -BM- MSCs-NaHS-CM**	**434.6 ± 6.5**^**bef**^	**71.9 ± 1.3**^**bef**^	**112.3 ± 2.3**^**bcef**^

Data are represented as Means ± S.E of 8 rats/group.

^a^Significant change at P <0.05 in comparison with the control group.

^b^Significant change at P <0.05 in comparison with the untreated CIA group.

^c^Significant change at P <0.05 in comparison with the CIA + naproxen group.

^d^Significant change at P <0.05 in comparison with the CIA + BM-MSCs group.

^e^Significant change at P <0.05 in comparison with the CIA + BM-MSCs-CM group.

^f^significant change at P <0.05 in comparison with the CIA + NaHS group.

### Gene expression findings

**Fig. 5:**
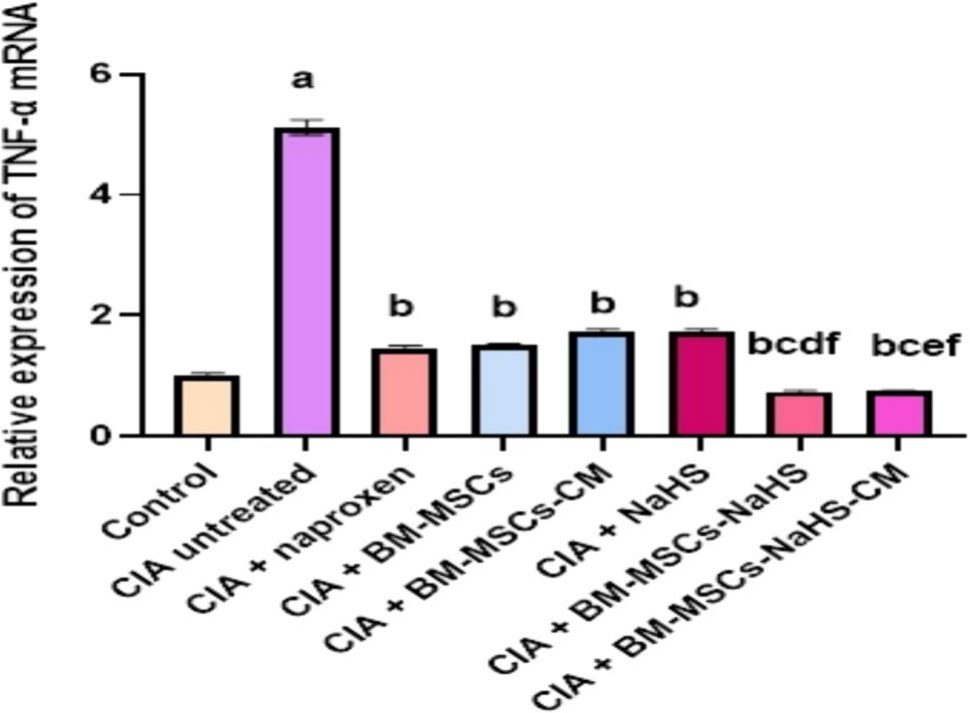
Effect of different treatments on synovial membrane TNF-α mRNA expression level in CIA rats. Data are represented as means ± S.E of 6 rats/group. ^a^Significant change at *P* <0.05 in comparison with the control group. ^b^Significant change at *P* <0.05 in comparison with the untreated CIA group. ^c^Significant change at *P* <0.05 in comparison with the CIA + naproxen group. ^d^Significant change at *P* <0.05 in comparison with the CIA + BM-MSCs group. ^e^Significant change at *P* <0.05 in comparison with the CIA + BM-MSCs-CM group. ^f^Significant change at *P* < 0.05 in comparison with the CIA + NaHS group

Figure 5 illustrates the impact of various treatments on the expression level of TNF-α mRNA in the synovial membrane of CIA rats. The injection of collagen type II resulted in a significant (*P* > 0.05) upregulation of TNF-α mRNA expression compared to the control group. However, the offered treatments for the CIA rats evoked a significant (*P* > 0.05) downregulation of TNF-α mRNA expression *versus* the untreated CIA rats. Specifically, the group treated with BM-MSCs-NaHS showed a significant (*P* > 0.05) downregulation of TNF-α mRNA expression compared to those treated with naproxen, BM-MSCs, or NaHS alone. Similarly, the group treated with BM-MSCs-NaHS-CM exhibited a significant (*P* > 0.05) downregulation of TNF-α mRNA expression compared to those treated with naproxen, BM-MSCs-CM, or NaHS alone.

### Protein expression outcomes

**Fig. 6:**
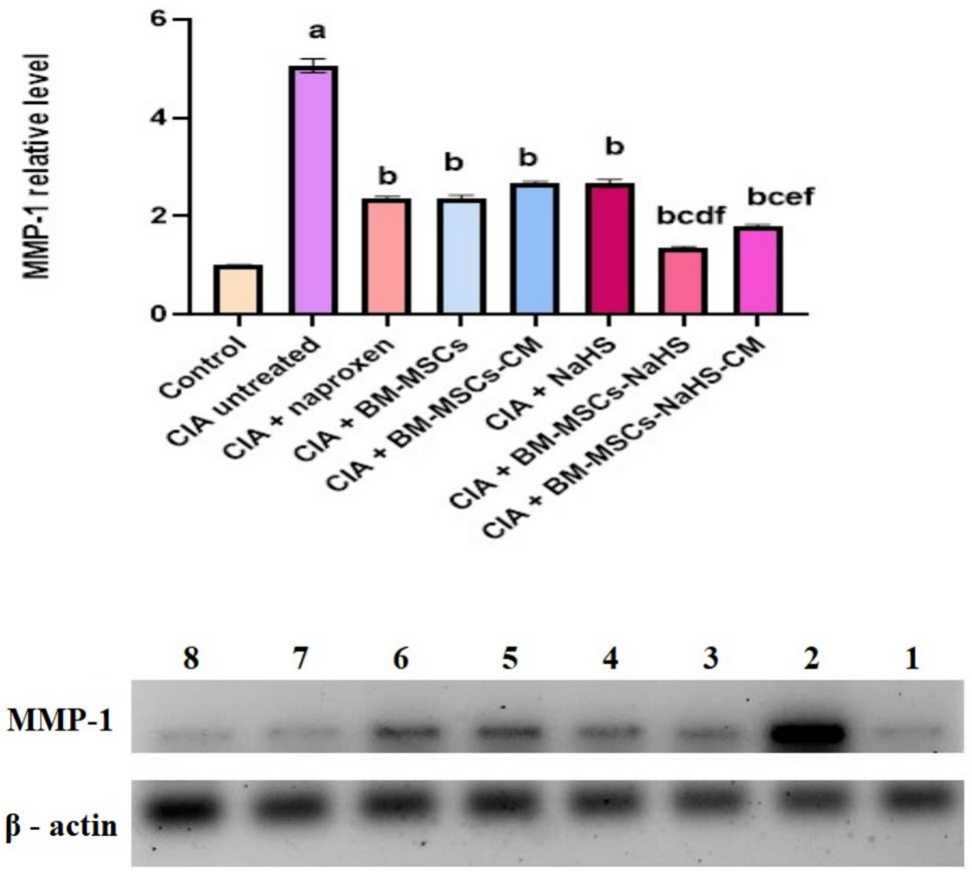
Effect of different treatments on synovial membrane MMP-1 protein expression level in CIA rats. (1) Control, (2) CIA untreated, (3) CIA + naproxen, (4) CIA + BM-MSCs, (5) CIA + BM-MSCs-CM, (6) CIA + NaHS, (7) CIA + BM-MSCs-NaHS, and (8) CIA + BM-MSCs-NaHS-CM. Data are represented as means ± S.E of 6 rats/group. ^a^Significant change at *P* <0.05 in comparison with the control group. ^b^Significant change at *P* <0.05 in comparison with the untreated CIA group. ^c^Significant change at *P* <0.05 in comparison with the CIA + naproxen group. ^d^Significant change at *P* <0.05 in comparison with the CIA + BM-MSCs group. ^e^Significant change at *P* <0.05 in comparison with the CIA + BM-MSCs-CM group. ^f^Significant change at *P* < 0.05 in comparison with the CIA + NaHS group

Figure 6 illustrates the impact of various treatments on the expression of MMP-1 protein in the synovial membrane of CIA rats. Injection of collagen type II resulted in a significant (*P* > 0.05) elevation in MMP-1 protein expression compared to the control group. However, all treatments administered to the CIA rats caused a significant (*P* > 0.05) reduction of MMP-1 protein expression relative to the untreated CIA rats. Infusion of BM-MCs-NaHS in CIA rats led to a significant (*P* > 0.05) decline in MMP-1 protein expression *versus* those treated with naproxen, BM-MSCs, or NaHS alone. Additionally, treatment with BM-MSCs-NaHS-CM resulted in a significant (*P* > 0.05) decrease in MMP-1 protein expression compared to those treated with naproxen, BM-MSCs-CM, or NaHS alone.

### Histological findings

**Fig. 7:**
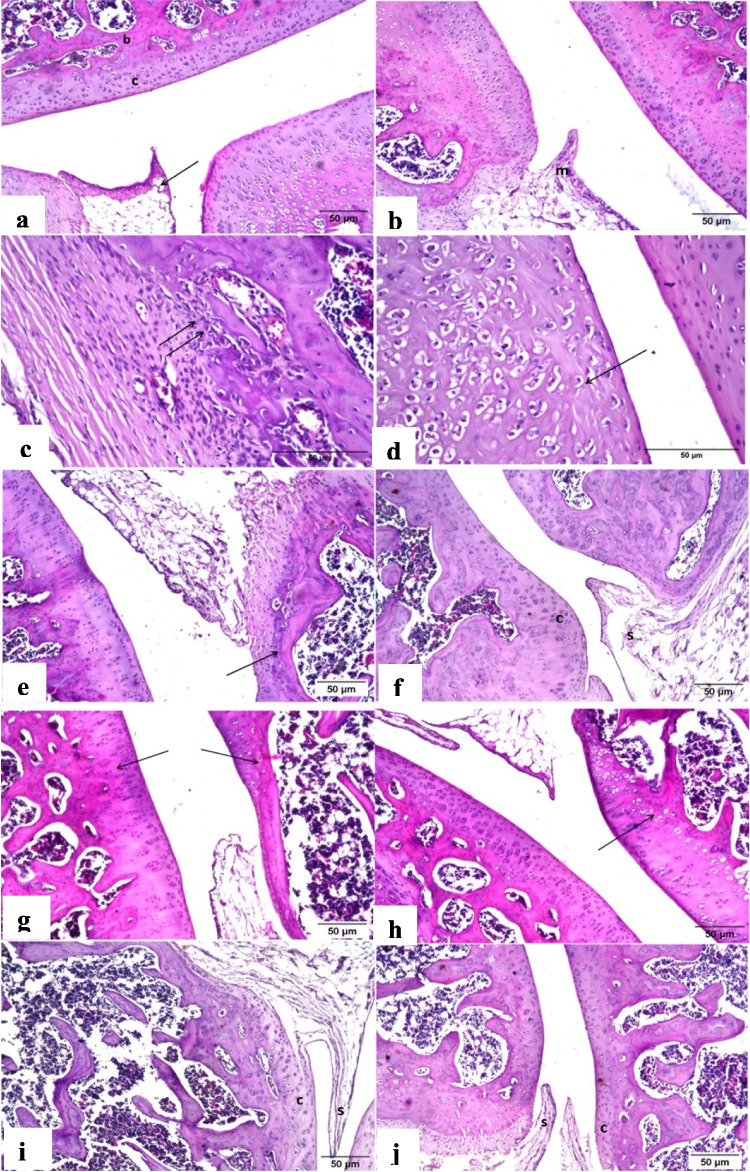
Representative light microscopy images of H&E stained knee joints section of rat in** a** control group exhibiting intact histological structure of the synovial membrane (arrow) articular cartilage (c) and underlying bone (b), **b** CIA untreated group exhibiting inflammatory cells infiltration in the wall of synovial membrane (m), **c** CIA untreated group showing cellular degeneration in the articular cartilaginous surface, **d** CIA untreated group displaying cellular degeneration and compression in the underlying bony structure, **e** CIA rat treated with naproxen showing normal histological structure of the synovial membrane (s) with degeneration and desquamation of articular cartilaginous surface in focal manner (arrow), **f** CIA rat infused with BM-MSCs showing normal histological structure of synovial membrane (s) and cartilaginous surface (c) of the knee joint, **g** CIA rat infused with BM-MSCs-CM showing hyalinization and atrophy of articular cartilaginous surface (arrow), **h** CIA rat treated with NaHS showing degeneration and hyalinization of cartilaginous articular surface (arrow), **i** CIA rat infused with BM-MSCs-NaHS showing intact histological structure of both synovial membrane (s) and cartilaginous surface (c), and **j** CIA rat infused with BM-MSCs-NaHS-CM exhibiting intact histological structure of synovial membrane (s) and cartilaginous surface (c) (scale bar 50 μm)

Histological examination of knee joint tissue section of rat from the control group revealed that there is no histopathological alteration in the synovial membrane and articular cartilaginous surface (Fig. 7a). Nevertheless, histological examination of knee joint tissue section of rat in the untreated CIA group indicated the presence of inflammatory cells infiltration in the synovial membrane (Fig. 7b). The articular cartilaginous surface showed cellular degeneration (Fig. 7c) while the underlying bony structure showed cellular degeneration and compression (Fig. 7d).

Histological finding of knee joint tissue section of CIA rat treated with naproxen showed that there is no histopathological alteration in the synovial membrane while the articular cartilaginous surface showed focal degeneration and cellular desquamation (Fig. 7e). Meanwhile, histological examination of knee joint tissue section of CIA rat infused with BM-MSCs indicated that there are no histopathological alterations in both synovial membrane and articular cartilaginous surface (Fig. 7f). The histological observation of knee joint tissue section of CIA rat infused with BM-MSCs-CM revealed that there are hyalinization and atrophy in the wall of articular cartilaginous surface while the synovial membrane was intact (Fig. 7g).

Histological examination of knee joint tissue section of CIA rat administered with NaHS showed cellular degeneration and hyalinization in the wall of articular cartilaginous surface while the synovial membrane was intact (Fig. 7h). However, histological finding of knee joint tissue section of CIA rats infused with BM-MSCs-NaHS or BM-MSCs-NaHS-CM indicated that there are no histopathological alterations in both synovial membrane and articular cartilaginous surface (Fig. 7i and j respectively).

## Discussion

Rheumatoid arthritis (RA) is a chronic autoimmune condition that mainly targets the joints, resulting in inflammation, discomfort, and possible joint damage. Timely use of affordable and effective treatments for rheumatoid arthritis is essential to reduce its global impact (Black et al. 2023). In recent years, mesenchymal stem cells (MSCs) have emerged as a promising therapeutic option for autoimmune diseases due to their unique immunomodulatory properties, which offers new hope for RA treatment. Research suggests that enhancing the efficacy of bone marrow-derived mesenchymal stem cells (BM-MSCs) through strategies such as cytokine priming or combination with biological agents can significantly improve treatment outcomes, potentially providing more effective long-term solutions for patients with RA (Sarsenova et al. 2021b).

This study was designed to investigate the potential of NaHS as an H_2_S donor in enhancing the therapeutic efficacy of BM-MSCs in treating arthritis in the knee joints of CIA model in rats.

The BM-MSCs used in this study were obtained from male rats and were characterized by their adhesiveness, fusiform shape, and expression of surface markers CD44^+^ and CD73^+^, while lacking the hematopoietic lineage marker CD34^−^ (Aglan et al. 2024b; Nazemian et al. 2018). Positive Prussian blue staining confirmed the presence of BM-MSCs and BM-MSCs preconditioned with NaHS in damaged knee joints, demonstrating successful engraftment following infusion. Recent studies indicate that BM-MSCs possess the ability to migrate toward inflamed tissues, integrating into the joint microenvironment and contributing to tissue repair and regeneration (Fu et al. 2019; Mostafa et al. 2021). Recent research has shown that mesenchymal stem cells (MSCs) actively express a diverse range of chemokines and their corresponding receptors, forming an intricate chemotactic network. This network plays a crucial role in guiding circulating cells to sites of injury while also facilitating the recruitment of immune cells to inflamed tissues. Furthermore, activated MSCs secrete key monocyte-attracting chemokines, including CCL2, CCL3, CXCL2, and CCL12, which enhance monocyte migration to affected areas. This chemokine-mediated signaling is fundamental to MSC-driven immunomodulation and tissue repair (Cuesta-Gomez et al. 2021; Harrell et al. 2021; Han et al. 2022).

While animal models cannot fully replicate the complexity of human rheumatoid arthritis, they are instrumental in studying disease mechanisms and evaluating potential therapies. Among these, the collagen-induced arthritis model is widely utilized due to its resemblance to human RA in clinical symptoms, tissue changes, and immune responses. In this model, arthritis is induced in genetically susceptible mice or rats by immunization with type II collagen emulsified in complete Freund’s adjuvant, leading to synovial inflammation, pannus formation, cartilage degradation, and bone erosion—hallmarks of RA (Kong et al. 2023; Abdallah et al. 2025). Pain is a prominent characteristic of inflammation, as described by Celsus in the first century (Gilroy and Bishop-Bailey 2019). The transmission of acute pain signals in response to noxious stimuli includes the activation of specialized sensory neurons called nociceptors by inflammatory triggers (Yam et al. 2018). Innate immune cells, including neutrophils, mast cells, and macrophages, contribute to pain and its sensitization by releasing inflammatory mediators. Neutrophils produce cytokines that amplify nociceptive signaling, while mast cells release histamine, directly stimulating pain receptors. Macrophages further sustain inflammation by secreting pro-inflammatory cytokines such as TNF-α and interleukins, prolonging pain sensitivity (Cunha et al. 2008; Ji et al. 2018). Pro-inflammatory lipids, including cyclooxygenase (COX)-dependent molecules like prostanoids (prostaglandins, prostacyclins, and thromboxanes), are known for their role in causing pain and pain sensitization (Gunaydin and Bilge 2018). Additionally, pro-inflammatory cytokines such as IL-1β, IL-6, TNF-α, IL-17 A, and IL-5 represent another crucial class of stimuli that activate nociceptors and promote pain sensitization (Pinho-Ribeiro et al. 2017). Injection of type II collagen significantly impaired locomotion in rats, as indicated by increased gait scores and heightened paw withdrawal frequency. Recent studies using collagen-induced arthritis (CIA) models have reported similar gait disturbances, confirming their relevance in assessing arthritis progression and therapeutic interventions (Orhan et al. 2021; Wang et al. 2021b).

In our study, we observe a reduction in pain among rats following treatment with naproxen, BM-MSCs, BM-MSCs-CM, NaHS, BM-MSCs-NaHS, or BM-MSCs-NaHS-CM, as evidenced by a decrease in gait score and PWF. The observed improvements in pain behavior and reduced gait scores in CIA rats treated with naproxen are consistent with recent findings from Katri et al. (2019) and Paglia et al. (2021). The analgesic effect of naproxen may stem from its inhibition of prostaglandin (PG) synthesis. While PGs do not directly induce pain, they sensitize peripheral nociceptors to other mediators like histamine and bradykinin, thereby enhancing pain perception. For instance, prostaglandin E₂ (PGE₂) lowers the activation thresholds of nociceptive nerve endings, contributing to increased sensitivity to mechanical and thermal stimuli (Jang et al. 2020).

The ability of BM-MSCs to reduce gait score and pain in CIA rats may stem from their ability to inhibit inflammation via interactions with immune cells and paracrine mechanisms (Sarsenova et al. 2021a). BM-MSCs can inhibit the maturation of dendritic cells (DCs), which play a role in stimulating inflammation by presenting antigens to autoreactive T cells; this leads to the production of cytokines that promote T-helper differentiation. Additionally, BM-MSCs boost DC generation through decreasing toll-like receptor (TLR) promotion and inhibiting IL-12 production from DCs (Shi et al. 2018).

The effectiveness of sodium hydrosulfide (NaHS) in reducing pain and joint swelling associated with joint inflammation has been demonstrated by Wang et al. (2021a). In their study, they found that NaHS pretreatment in IL-1β-induced chondrocytes significantly attenuated inflammatory cytokine overproduction and improved the balance between anabolic and catabolic activities, via inhibition of the PI3 K/Akt/NF-κB pathway. Additionally, Lin et al. (2014) showed that NaHS administration alleviated chronic neuropathic pain in rats by inhibiting the expression of the phosphorylated cyclic-AMP response element-binding (pCREB) in the spinal cord.

The significant improvement in walking ability and reduction of joint pain in CIA rats treated with BM-MSCs preconditioned with NaHS can be attributed to the enhanced therapeutic effects of BM-MSCs after preconditioning with NaHS on neuronal injury and neurological recovery, as highlighted by Zhang et al. (2016). These researchers provided insights into the potential mechanisms underlying the beneficial effects of H_2_S-preconditioned BM-MSCs, suggesting that they may enhance BM-MSC survival and promote the secretion of paracrine factors and protective cytokines, ultimately leading to improved outcomes.

Additionally, Nazemian et al. (2018) demonstrated that treatment with conditioned media from BM-MSCs can effectively reduce inflammation and alleviate pain behavior in arthritis. Their findings suggest that BM-MSCs-CM exerts a direct effect on inhibiting intracellular signaling pathways and pro-inflammatory cytokines, contributing to its therapeutic efficacy. Furthermore, previous studies have shown that injection of MSC-CM increases the expression of anti-inflammatory cytokines, decreases the expression of pro-inflammatory cytokines, and attenuates the activity of intracellular signaling pathways. As a result, these immune-modulatory actions alleviate spinal neuroinflammatory symptoms (Platas et al. 2013; Wang et al. 2016; Yew et al. 2011).

The data from our current study revealed that injection of collagen type II in rats led to increased serum levels of CRP, RF, and 14-3-3η. These findings are strongly supported by the research of Haikal et al. (2019) and Wang et al. (2020). CRP, a prototypical acute phase protein, plays a crucial role in the host’s defense mechanisms against infectious agents and in the inflammatory response. It is expressed by various cell types including smooth muscle cells, macrophages, endothelial cells, lymphocytes, and adipocytes (Sproston and Ashworth 2018). Serum CRP levels are frequently used as a prognostic marker for progressive joint damage and dysfunction. The binding of CRP to immunoglobulin Fc gamma receptors (FcgR) motivates the generation of pro-inflammatory cytokines and intensifying the inflammatory response (Newling et al. 2019). Studies have demonstrated a significant correlation between serum CRP levels and tissue inflammation scores from knee synovium biopsy samples in patients with RA (Orr et al. 2018). Kim et al. (2015) proposed that elevated levels of cytokines and TNF-α stimulate CRP production and release.

Serum RF is widely recognized as the serological hallmark of RA (Newkirk 2002) and has been consistently identified as the most reliable predictor of disease severity in numerous studies. Patients who test positive for RF typically experience more severe symptoms compared to those with negative results. RF plays a crucial role in the pathogenesis of RA by activating the classic complement pathway (IgM and IgG) and is highly prevalent in the synovial tissue of affected joints. The production of high-affinity RF in RA patients leads to T-cell activation and contributes to the inflammatory process by forming immune complexes. This, in turn, stimulates the production of pro-inflammatory cytokines such as TNF-α and IL-1β, further perpetuating chronic inflammation and bone destruction (van Delft and Huizinga 2020).

The 14-3-3 family of conserved regulatory proteins consists of seven isoforms: α/β, γ, δ/ζ, ε, η, θ/τ, and σ. In a study by Chavez-Muñoz et al. (2009), significantly higher levels of 14-3-3 η protein were observed in the synovial fluid and serum of arthritis patients compared to healthy individuals. The researches also noted a positive correlation between 14-3-3 η and matrix metalloproteinases (MMPs), suggesting its potential relevance to cartilage and bone destruction by regulating MMP expression in RA patients. Furthermore, Wang et al. (2020) proposed that 14-3-3 η may perpetuate inflammation by inducing factors such as IL-6 and exacerbate joint destruction via MMPs and RANKL.

The existing data suggests that treating CIA rats with the proposed interventions results in decrease in serum CRP, RF, and 14-3-3η levels. This decrease in serum CRP levels following naproxen treatment is consistent with the findings of Tarp et al. (2012). Previous studies have shown that naproxen can inhibit NF-κB activation in IL-1β-induced chondrocytes (Cheleschi et al. 2015) and reduce the production of the pro-inflammatory cytokine IL-6 by synovial tissue fibroblasts (Pelletier et al. 1997). Additionally, naproxen primarily works by inhibiting prostaglandins and cyclooxygenases enzyme activity (Brutzkus et al. 2023) which helps to suppress inflammation and decrease the levels of inflammatory markers observed in this study.

The impact of BM-MSCs infusion on serum CRP and RF levels in CIA rats is consistent with findings reported by El-Denshary et al. (2014) and Moghaddam et al. (2023). BM-MSCs have been shown to modulate the cytokine secretion profile of immune cells, resulting in increasing secretion of anti-inflammatory cytokines such as IL-4 and IL-10, while decreasing the secretion of pro-inflammatory mediators like TNF-α and interferon-c (IFN-c). Additionally, BM-MSCs have the ability to inhibit the promotion of M1-type macrophages and promote the production of M2-type macrophages. These M2-type macrophages exhibit heightened phagocytic activity and secrete Th2 cytokines, while simultaneously reducing levels of Th1 cytokines, including IL-1β, IFN-c, TNF-α, and IL-12. This modulation of the immune response by BM-MSCs suggests an enhancement of Th2 signaling, directly inhibiting the Th1 cascade reaction and thereby suppressing the propagation of inflammation. These findings provide valuable insights into the potential efficacy of BM-MSCs as antiarthritic and anti-inflammatory agents for managing RA (Ahmed et al. 2021).

The anti-inflammatory properties of NaHS have been demonstrated through a decrease in serum CRP, RF, and 14-3-3η levels in CIA rats after treatment with NaHS. This is attributed to its ability to reduce the expression of MMPs at sites of damage (Pan et al. 2016); Vela-Anero et al. 2017) and inhibit gonarthrosis (Aytekin et al. 2018). Furthermore, NaHS has been shown to induce cytoprotective mechanisms in the joint (Dief et al. 2015; Wu et al. 2016) and suppress the production of pro-inflammatory mediators (Sieghart et al. 2015). The significant reduction in serum CRP, RF, and 14-3-3η levels in CIA rats infused with BM-MSCs preconditioned with NaHS can be attributed to the enhancing effect of NaHS on the therapeutic efficacy of BM-MSCs against the inflammatory reactions associated with RA (Zhang et al. 2016).

The administration of BM-MSCs-CM or BM-MSCs-NaHS-CM to CIA rats decreased serum CRP, RF, and 14-3-3η levels significantly. This can be attributed to the immunomodulatory effects of CM, which alters the functions of B and T lymphocytes (Platas et al. 2013). Additionally, CM has been shown to alleviate inflammatory symptoms through its trophic factors (Nazemian et al. 2018). These findings suggest that CM has potential as a treatment for CIA.

The current findings suggest that injecting collagen type II in rats significantly increases the expression levels of TNF-α mRNA in the synovial membrane, which is consistent with the results reported by (Wei et al. 2004). TNF-α is produced by synovial macrophages, B cells, and NK cells and plays a crucial role in joint inflammation in RA (Brennan and McInnes 2008). It is highly present in most arthritic biopsies, and its overexpression can lead to spontaneous inflammation in various rodent arthritis models (Ma et al. 2019). TNF-α also contributes to cartilage degradation and bone resorption (Goldring 2003) and enhances RANK-L secretion by osteocytes, promoting osteoclastogenesis (Marahleh et al. 2019). Furthermore, TNF-α can directly stimulate the differentiation of monocyte/macrophage lineage cells into osteoclasts through a RANK-L-independent mechanism (Azuma et al. 2000; Lam et al. 2000). Another significant role of TNF-α in RA pathogenesis is its ability to stimulate the production of other inflammatory cytokines, such as IL-1β and IL-6, which attract leukocytes and create an inflammatory environment in the synovium (Brennan and McInnes 2008).

The treatment of CIA rats with naproxen, BM-MSCs, BM-MSCs-CM, NaHS, BM-MSCs-NaHS, or BM-MSCs-NaHS-CM resulted in a significant decrease in TNF-α mRNA expression levels in the synovial membrane. The downregulation of TNF-α gene expression due to naproxen treatment is consistent with the findings of Dief et al. (2015), who reported that naproxen suppresses NF-κB activity, leading to reduce in TNF-α levels.

Similarly, the observed decrease in TNF-α gene expression levels after BM-MSC infusion in CIA rats aligns with the findings of Liu et al. (2010). Their study showed that BM-MSCs inhibit the proliferation of TNF-α-stimulated fibroblast-like synoviocytes, as well as stimulated T-cell proliferation and the downregulation of pro-inflammatory cytokine production, including TNF-α. In terms of the effect of NaHS on TNF-α gene expression, Guo et al. (2014) and Sen et al. (2012) have reported that NaHS has anti-inflammatory properties by inhibiting NF-κB p65 activation, a crucial pro-inflammatory factor in RA. H_2_S has been shown to induce persulfidation of the p65 subunit of NF-κB, resulting in its retention in the cytosol, reduced NF-κB DNA binding, and inhibition of NF-κB-related inflammatory molecule production (Sen et al. 2012).

In line with the findings of Nazemian et al. (2018) and Shi et al. (2022), treating CIA rats with BM-MSCs-CM or BM-MSCs preconditioned with NaHS-CM resulted in a significant decrease in TNF-α mRNA expression levels in the synovial membrane. Jin et al. (2022) also reported that MSC-CM treatment not only reduces TNF-α secretion but also increases the secretion of anti-inflammatory cytokines at the site of inflammation. Additionally, their research showed that the treatment with MSC-CM inhibits the phosphorylation of mitogen-activated protein kinases (MAPKs) and nuclear factor kappa-B (NF-κB) in lipopolysaccharide (LPS)-stimulated RAW264.7 cells. This inhibition leads to a reduction in the secretion of pro-inflammatory cytokines such as TNF-α and IL-6, while promoting the expression of anti-inflammatory cytokines like IL-10.

The data from our current study shows a significant increase in MMP-1 protein levels in the synovial membrane of rats after collagen type II injection. This finding is consistent with previous studies by Heard et al. (2012) and Araki and Mimura (2017), which also observed an upregulation of MMP1 expression in synovial tissue in RA. It has been documented that, various cytokines and chemokine, such as IL-6/TNF-α and granulocyte colony-stimulating factors, can stimulate neovascularization, leukocyte migration, and the expansion of synovial fibroblast-like cells (FLS) (Alivernini et al. 2022). These FLS cells then secrete MMP-1, which contributes to its upregulation, as seen in our study (Araki and Mimura 2017; Heard et al. 2012).

The administration of the proposed treatments led to a significant decrease in MMP-1 protein levels in the synovial membrane of CIA rats. This decrease in MMP-1 protein levels in CIA rats treated with naproxen is consistent with the findings of Sadowski and Steinmeyer (2001), who studied the effects of various non-steroidal anti-inflammatory drugs (NSAIDs) on collagenases in bovine chondrocytes stimulated with IL-1 alpha. Their research showed that indomethacin, meloxicam, and naproxen reduced IL-1-stimulated collagenolytic activity by downregulating the expression of the MMP-1 gene. The downregulatory effect of naproxen on MMP-1 levels is primarily due to its ability to suppress the synthesis of prostaglandin (PGE_2_), which is known to induce the production and secretion of MMP-1 in both rat and human osteoblast cells. This anti-inflammatory mechanism of naproxen involves inhibiting the enzymatic activity of COX enzymes, which is the initial step in the synthesis of PG from arachidonic acid (Brutzkus et al. 2023).

The impact of BM-MSCs or their CM on MMP-1 protein expression is associated with their ability to suppress Th17 differentiation. This suppression occurs through the upregulation of various cytokines and chemokines, such as IL-10 and CCL2, while simultaneously inhibiting the STAT3 pathway (Liu et al. 2015). Inhibition of the STAT3 pathway leads to reduced Th17 differentiation by down-regulating IL-17 expression (Lee et al. 2025), which is known to play a role in MMP-1 activation (Cortez et al. 2007). This ultimately results in the inhibition of MMP-1 production by synoviocytes.

The downregulation of MMP-1 protein expression due to NaHS, an H_2_S donor, is supported by Pan et al. (2016). They demonstrated that NaHS reduces the expression of MMPs at sites of cartilage damage. Furthermore, NaHS has been shown to induce cytoprotective systems within the joint (Wu et al. 2016) and reduce the production of pro-inflammatory mediators, such as IL-6 (Kloesch et al. 2010; Sieghart et al. 2015). These mediators play a crucial role in the hyperproliferation of FLS and their subsequent production of MMPs (Alivernini et al. 2022).

Histological examination of knee joint tissues from untreated collagen-induced arthritis (CIA) rats reveals significant changes, including infiltration of inflammatory cells into the synovial membrane, degeneration of the articular cartilage, and damage to the underlying bone structure. These observations are in line with recent studies that have shown similar pathological alterations in CIA models, including synovial hyperplasia, cartilage erosion, bone damage, synovitis, pannus formation, and a significant influx of inflammatory cells into the sub-synovial connective tissue(Babaahmadi et al. 2023; Hegen et al. 2008). Synovitis is a condition characterized by inflammation of the joint capsule, which includes the synovial membrane, synovial fluid, and the respective bones (Aletaha and Smolen 2018). This inflammation is caused by a complex interaction between various types of immune cells, such as dendritic cells, T cells, macrophages, B cells, neutrophils, fibroblasts, and osteoclasts. In individuals with RA, the presence of specific autoantigens continuously activates these immune cells, leading to a chronic state of inflammation in the joint (Firestein and McInnes 2017). This chronic inflammation causes the synovial membrane to expand, forming a tissue called “pannus,” which invades the surrounding bone at the junction between cartilage and bone. Over time, this can result in bone erosion and degradation of cartilage (Aletaha and Smolen 2018).

Histological examination of knee joint tissue section of CIA rats treated by naproxen showed smooth undamaged synovial membrane without positive effect on the articular cartilage. This observation is in consistent with that of Dief et al. (2015). Mechanistically, naproxen, like other NSAIDs, possesses the property of blocking the production of prostaglandins (Crofford 2013), which are responsible for cartilage destruction and bone loss (Kim and Kim 2005).

Histological assessment of knee joint tissue sections from CIA rats that were treated with BM-MSCs or their conditioned media showed regeneration of synovial membrane cells. This observed regeneration is consistent with previous studies (Kim et al. 2001; Lee et al. 2018; Mao et al. 2010). BM-MSCs have the ability to regulate various types of immune cells through the expression of immunomodulators, including DCs, monocytes/macrophages, regulatory B cells, regulatory T cells, Th1, Th2, Th17, natural killer (NK) cells, natural killer T (NKT) cells, innate lymphoid cells (ILCs), myeloid-derived suppressor cells (MDSCs), neutrophils, and mast cells. The mechanism by which BM-MSCs suppress immune reactions involves both the expression of immunomodulatory substances and direct cell contact (Jiang and Xu 2020), which may contribute to the greater effectiveness of BM-MSCs compared to their conditioned media in tissue recovery during inflammation.

In the current study, histological evaluation of knee joint tissue sections from CIA rats treated with NaHS showed evidence of synovial membrane cell regeneration. This finding is consistent with previous studies by Whiteman et al. (2010), Burguera et al. (2014), and Zhang et al. (2018). Additionally, degeneration and hyalinization were observed in the articular cartilage surface. The reparative effects of NaHS, as an H_2_S donor are attributed to its ability to upregulate Nrf2 protein expression and transcription of two key antioxidant genes, peroxiredoxin-1 and NAD(P)H dehydrogenase quinone 1, in osteoclast precursors (CD11b + human monocytes). NaHS has also been shown to inhibit osteoclast differentiation (Gambari et al. 2014). Furthermore, NaHS activates the epigenetic enzyme histone deacetylase 3 (HDAC3), downregulates inflammatory signaling, and maintains sulfhydration of runt-related transcription factor 2 (RUNX2), ultimately improving osteoclastogenesis (Behera et al. 2018).

The histological examination of knee joint tissue sections from CIA rats, infused with BM-MSCs-NaHS or BM-MSCs-NaHS-CM, revealed intact synovial membranes and articular cartilaginous surfaces. The combined therapy (BM-MSCs + NaHS) compared to NaHS alone showed improvement after treatment, which may be indirectly attributed to the upregulation of BM-MSC survival and secretion of paracrine factors (Zhang et al. 2016).

These findings highlight the potential therapeutic benefits of using BM-MSCs preconditioned with NaHS compared to using BM-MSCs or NaHS alone in treating rats with CIA. Additionally, the use of BM-MSCs preconditioned with NaHS-CM showed even greater therapeutic effects compared to using BM-MSCs-CM alone. This was demonstrated by Zhang et al. (2016) who found that H_2_S protects BM-MCs from apoptosis by regulating the Bax/Bcl-2 ratio. This protective effect is likely mediated by the activation of the PI3 K/Akt signaling pathway, which leads to increased levels of Bcl-2 protein and inhibition of the cytochrome c-caspase-3/9 apoptosis pathway, ultimately promoting cell survival (Zhang et al. 2013). Furthermore, H_2_S was found to enhance the proliferation of BM-MCs through the Akt and ERK1/2 pathways, which play important roles in various cellular functions such as survival, proliferation, differentiation, intracellular trafficking, and motility (Rauch et al. 2011; White et al. 2000).

## Conclusion

In conclusion, this study provides scientific evidence supporting the use of NaHS in the conditioned media of BM-MSCs for the treatment of CIA. The addition of NaHS has been shown to enhance the anti-inflammatory potential, immunomodulatory action, and regenerative capacity of BM-MSCs, making it a promising strategy for treating patients with RA. These findings suggest that NaHS could serve as a valuable adjunct in stem cell-based therapies, offering potential for improved clinical outcomes in the management of RA. Further investigations into the mechanisms underlying these effects could provide additional insights into the therapeutic potential of NaHS in autoimmune and inflammatory diseases.

## Data Availability

The datasets used and/or analyzed during the current study are available from the corresponding author on reasonable request.
